# Developing a measure of mental health service satisfaction for use in low income countries: a mixed methods study

**DOI:** 10.1186/s12913-017-2126-2

**Published:** 2017-03-09

**Authors:** Rosie Mayston, Kassahun Habtamu, Girmay Medhin, Atalay Alem, Abebaw Fekadu, Alehegn Habtamu, Martin Prince, Charlotte Hanlon

**Affiliations:** 1 King’s College London, Institute of Psychiatry, Psychology and Neuroscience, Centre for Global Mental Health, London, UK; 2 Addis Ababa University, School of Psychology, Addis Ababa, Ethiopia; 3 Department of Psychiatry, Addis Ababa University, College of Health Sciences, School of Medicine, PO 9086, Addis Ababa, Ethiopia; 4 Addis Ababa University, Aklilu-Lemma Institute of Pathobiology, Addis Ababa, Ethiopia; 5 Department of Psychological Medicine, Centre for Affective Disorders, King’s College London, Institute of Psychiatry, Psychology and Neuroscience, London, UK

**Keywords:** Service satisfaction, Mental health services, Community mental health services, Primary health care, Measurement, sub-Saharan Africa, Ethiopia, Mental health

## Abstract

**Background:**

Service satisfaction is integral to quality of care and measures are therefore considered important indicators of quality. Patient’s responses to their experiences of using services are under-researched in the context of mental healthcare in low income countries. Our aim was to use mixed methods to develop a new measure of satisfaction for use among consumers of the new models of mental healthcare which are currently being scaled-up.

**Methods:**

We used qualitative methods to explore the concept of service satisfaction. On the basis of these findings, we developed a new ‘Mental health service satisfaction scale’ (MHSSS v0.0) by adapting existing measures of service satisfaction. We evaluated psychometric properties of the new measure, among a sample of service users with severe mental disorder (SMD) (*n* = 200) and caregivers (*n* = 200). Following expert review, a modified version of the measure was developed (MHSSS v1.0) and psychometric properties were examined with data from a second independent sample (*n* = 150 service users with SMD and *n* = 150 caregivers).

**Results:**

Factors identified in analysis of the first quantitative sample coincide with core concepts of service satisfaction as reported in the literature and were reflected in the key themes which emerged from our qualitative study: interpersonal factors, efficacy, communication, technical competency and adequacy of facilities. There was generally consensus among caregivers and service users regarding dimensions of satisfaction. However there was evidence of some differences in prioritization. Revisions made to version 0.0 of the Mental Health Service Satisfaction Scale (MHSSS) led to an improved instrument, with excellent internal consistency, convergent validity and factor loadings indicative of a uni-dimensional construct.

**Conclusions:**

Our findings suggest that conceptions of service satisfaction among people accessing a service for SMD are broadly similar with those established in the literature. Our findings indicate that the MHSSS might be a useful candidate for inclusion in the new toolkit of measures needed to facilitate monitoring of service satisfaction which will be crucial to quality improvement.

**Electronic supplementary material:**

The online version of this article (doi:10.1186/s12913-017-2126-2) contains supplementary material, which is available to authorized users.

## Background

**Fig. 1:**
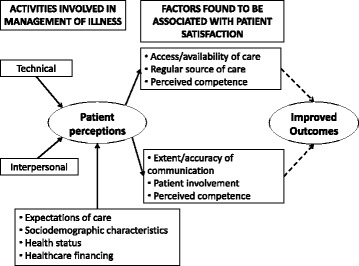
Conceptual framework- Service Satisfaction (based on Cleary et al. 1988 [1])

Service satisfaction has been described as a patient response to salient aspects of their experience of services [1] and as an outcome of care, particularly interpersonal processes [2]. Although service satisfaction should not be conflated with quality of care, policymakers and planners cannot afford to ignore subjective reactions to service use which are inherent within reported satisfaction. As described in the model of service satisfaction set out by Cleary (see Fig. 1), satisfaction is integral to quality of care: for example, one of the dominant predictors of satisfaction is positive perceptions of patient-physician communication [3]. In turn, accurate communication and patient involvement are integral to effective diagnosis and treatment. Patients who feel more involved in their care by physicians are more likely to be satisfied and therefore more likely to adhere to treatment and engage with care, achieving positive outcomes. Measures of service satisfaction can therefore be considered as a useful constituent measure of quality, with dissatisfaction potentially indicating less than optimal communication, lack of patient involvement, lack of engagement with patient preferences, lack of continuity or perceived problems with availability or technical competence [1].

The importance of the inclusion of service user perspectives in the development and evaluation of services has been recognised in psychiatric settings in high income countries [4–6] and satisfaction has been identified as one way of identifying service users’ perspectives on the care they receive. In the context of mental health care, dissatisfaction with care received has been found to be associated with adverse outcomes: lack of service uptake, poor therapeutic alliance, discontinuation of care, a higher number of unmet needs and lower quality of life [7–11]. However, consistently high levels of satisfaction reported in studies have prompted researchers to warn that caution needs to be exercised in the interpretation of results [12]. It is likely that in settings where consumers are disempowered or where there is a perceived or real lack of choice of services, or a perceived or real lack of preparedness/willingness of services to change, this perception is likely to influence reported satisfaction levels [13].

Efforts to close the mental health treatment gap in low and middle income country (LMIC) settings are continuing apace [14, 15]. Task-sharing, whereby non-specialist healthcare workers based in primary care are trained to deliver treatment for mental disorders while supported by specialists, has been adopted as the preferred approach to facilitate rapid scale-up of services [16–18]. The experiences of service users and caregivers of these new models of mental health care, including their satisfaction with services received are under-researched. There is some evidence that lack of experience of service utilization might limit the ability of people accessing new services in LMICs to critically appraise the care they receive [19, 20]. Nonetheless, it is essential that effective strategies for monitoring and evaluation, including measuring patient’s responses to services, are developed as an integral component of scale-up, prioritizing quality of care alongside coverage [21].

Work on measuring consumer satisfaction with services in LMICs has focused on satisfaction with general hospital care, primary care [22, 23] and family planning/HIV services, including one measure designed for use in Ethiopia with versions for general hospital and primary care [24]. We found no measures of service satisfaction or other aspects of consumer experience of mental health services developed for use in African or other low income country settings. The Verona Service Satisfaction Scale (VSSS) [25] remains the most widely used measure of satisfaction with mental health services in European settings, with findings from a number of studies supporting the construct validity of the instrument [26].

The aim of the current study was to explore the dimensions and meaning of satisfaction with services and develop a testable measure among users of a psychiatric nurse delivered out-patients service for people living with SMD. The current study took place in the context of preparatory work for the TaSCS study (Task-Sharing for the Care of Severe mental disorders in a low income country), a non-inferiority trial which seeks to test the impact of the delivery of care for people with severe mental disorders (SMD) by non-specialist healthcare workers in primary care as compared with the existing model [27].

## Methods

### Study design

We used a mixed methods study design to: a) identify the components of the service that contributed to satisfaction/dissatisfaction and assess the credibility of items included in existing scales; b) use the results of the qualitative study to make adaptations to existing scales, c) evaluate the psychometric properties and predictive validity of the measure in a large sample of service users and caregivers, d) refine the measure in light of findings from c), and e) evaluate the construct validity and psychometric properties of the refined scale in an independent sample of service users and caregivers.

### Setting

The study was carried out in Butajira town and the surrounding three districts, located 135 km south of Addis Ababa, the capital of Ethiopia, in the Gurage Zone of the Southern Nations, Nationalities and Peoples’ Region. As is the case in much of rural Ethiopia, the majority of the population lives by subsistence farming, with urban dwellers relying on trade as a key income generating activity. At the time of the study, the psychiatric nurse-led outpatient unit at the Butajira General Hospital was the only mental health service available for the surrounding districts. The nearest in-patient mental health service is in Addis Ababa.

### The qualitative study

The aim of the qualitative study was to explore which aspects of the mental health service might contribute to participant’s satisfaction with the service. Facilitators began the focus group discussions (FGDs) and in-depth interviews by asking participants what treatments/components of the service were most important to them. Participants were also asked to comment on the importance of factors included in existing measures of service satisfaction [24, 25]. Six in-depth interviews were carried out with service-users. Five FGDs were carried out (two with service users and three with caregivers, with 8 participants in each FGD, *n* = 40 in total). Participants were selected purposively to ensure that those living in both urban (Butajira town) and rural (surrounding districts) were represented in the qualitative study. The qualitative interviewer was a male research assistant with a Masters in clinical psychology. He was not known to participants. He had previous experience with qualitative work but was supervised by CH for this study. The audio-recordings of the FGDs and in-depth interviews were transcribed in Amharic and translated into English by interviewers prior to coding. Data were managed using Open Code 4.0 qualitative software [28]. We used a framework approach to analyse transcripts of FGDs [29].

### Quantitative study

The aim of our quantitative research was to explore the psychometric properties, validity and reliability of a locally relevant measure of service satisfaction. We carried out two studies. Firstly, we administered the measure of satisfaction compiled and adapted after the qualitative study to a sample of service users (*n* = 200) and their caregivers (*n* = 200). The quantitative component of the satisfaction scale study was carried out in parallel with a study which aimed to develop a locally appropriate measure of functioning for use among people living with SMD [30]. Sample sizes were calculated on the parameters necessary for the functioning scale, which allowed sufficient power for analyses related to the satisfaction instrument. Participants were consecutive attendees of the psychiatric clinic at Butajira Hospital who were returning to the service for a follow-up visit in March 2014. See Table 1 for characteristics of participants in both quantitative samples. Voluntary written informed consent was obtained from all participants (witnessed thumb print for those who were illiterate). Data were collected by experienced data collectors. All interviews were conducted in the hospital (in the project office or under the tree in the hospital grounds). Interviews took 10–15 min.

**Table 1:** Sample 1: Internal consistency and item-item correlations

	Service users		Caregivers	
Item	Item-test correlation	Average Item-item correlation	Item-test correlation	Average Item-item correlation
The health worker treated me with courtesy	0.55	0.29	0.50	0.23
The health worker listened to me carefully	0.65	0.28	0.55	0.23
The health worker explained me things in a way I understood	0.70	0.28	0.61	0.22
The health facility was clean	0.45	0.30	0.52	0.23
The waiting room was clean	0.53	0.29	0.54	0.23
The latrine was clean	0.47	0.29	0.54	0.23
The waiting time was acceptable	0.27	0.31	0.29	0.24
I have enough time to discuss with health worker	0.58	0.29	0.53	0.23
I was give information in a way I understood	0.80	0.27	0.70	0.22
I received helpful advice	0.72	0.28	0.54	0.23
Administrative staff treated me with courtesy and respect	0.50	0.29	0.51	0.23
The health worker involved my family helpfully	0.69	0.28	0.67	0.22
My privacy is respected	0.30	0.30	0.47	0.23
I have the opportunity for follow up with the same health worker	0.60	0.29	0.48	0.23
My personal information is kept confidential	0.49	0.29	0.38	0.24
Referral to specialist is possible	0.47	0.29	0.41	0.23
The service is effective at decreasing symptoms	0.63	0.28	0.53	0.23
The service is effective at decreasing relapses	0.68	0.28	0.51	0.23
The service is effective at helping with economic problems	0.63	0.28	0.49	0.23
It is possible to see the health worker when needed	0.67	0.28	0.59	0.22

For service users, overall Cronbach’s alpha = 0.89; For caregivers, overall Cronbach’s alpha = 0.85

The initial service satisfaction measure consisted of 20 items, all of which were phrased as positive statements about the service (health workers’ skills and attributes, facilities, impacts). These were made up of items from O-PAHC and VSSS which were confirmed as relevant to participants’ satisfaction with the service received in the qualitative study. Qualitative findings were used to shape appropriate adaptations to ensure that items included in the questionnaire were grounded in the experiences of local service users. For example, service users did not distinguish the type of health worker and so more generic terms were used than in the original scales (the O-PAHC items distinguish between doctors and nurses). It was also found to be necessary to add a specific item on the cleanliness of the waiting area. Questions were framed to ask participants about their last clinical encounter with the service. At the beginning of the questionnaire, the interviewer said: “thinking back to the last appointment you had at a health facility for mental health care, please tell me how much you disagree or agree with the following statements..”. There were four possible response options: “Strongly agree”; “Agree”; “Disagree” and “Strongly disagree”. Sociodemographic data were collected from all participants. Caregivers were asked to respond to questions with reference to their perception of the service user’s experience, attitudes and behaviours. In addition to the satisfaction measure, data were collected on adherence and therapeutic alliance, using the Morisky Medication Adherence Scale and the Helping Alliance Questionnaire [10, 31, 32]. To examine test-retest reliability, we randomly selected a sample of 50 participants with SMD and their caregivers from our sample and re-administered the scale within 7–10 days of the original administration.

After the first stage of the quantitative study, the instrument and study findings were reviewed by experts from the AFFIRM research consortium [33], consisting of epidemiologists, social scientists and clinicians with experience of working in sub-Saharan Africa. As a result of this meeting, the instrument was refined. The wording of four items about effectiveness which were originally worded “the service is effective at…” was revised to make them more specific, referring to “the treatment helped me to..” consistent with the way the rest of the questionnaire was framed, encouraging participants to refer to their most recent encounter when answering. Four additional questions were included, three related to how easy it was to access the service, as this was felt to be an important facet of service satisfaction that was particularly pertinent to the rural Ethiopian setting and not captured elsewhere in the questionnaire. A final item was added: “I would advise my family to come to the hospital” as this was felt to be a good indicator of overall satisfaction with the service. The revised version of the measure was administered to a second sample of service users (*n* = 150) and their caregivers (*n* = 150). The second sample was recruited in November 2014.

Analysis was carried out using STATA 11.0. Bartlett’s test of sphericity and Kaiser-Meyer-Olkin test (KMO) were carried out for each dataset in order to ensure suitability for factor analysis. Exploratory factor analysis (maximum likelihood extraction method and varimax rotation) was carried out using responses to the service satisfaction scales for sub-study 1 (version 0.0, service users and caregivers) and sub-study 2 (version 1.0, service users and caregivers). Factors with eigenvalues >1 were retained. For sub-study 1, internal consistency and stability (test-retest) were tested. In sub-study 2, internal consistency was tested. Three psychometric properties were assessed: item-test correlation; inter-item correlation and Cronbach’s alpha (internal consistency). Test-retest reliability was assessed using the kappa coefficient [34]. Finally, hypotheses relating to the predictive validity of the new measure were tested. We examined associations between mean satisfaction score and variables related to adherence and therapeutic alliance, carrying out multiple logistic regression and adjusting for sociodemographic variables found to be associated with mean service satisfaction scores in bivariate analysis.

### Ethics, consent and permission

Scale development was a component of preparatory work for the TaSCs trial [27]. Ethical approval for the preparatory study was obtained from the Institutional Review Board of the College of Health Sciences, Addis Ababa University (Reference Number 030/12/Psy).. Study participants were informed about the purpose of the study and written informed consent was secured from all participants prior to the start of data collection.

## Results

### Qualitative study

#### Benefits of treatment

Medication was identified as the single most important component of satisfaction with the service. Reduction in symptoms such as disordered sleep, anger and distress were reported as direct benefits by service users and caregivers. Important indirect benefits included: resuming education, getting married, becoming a parent, opening a shop, employment, reducing the burden upon caregivers. Medication and medication support prevented relapse and facilitated the continuation of these markers of “normal” life:

“*The treatment is very important now. It is helping us much. Those who discontinued the treatment by their own are found being shabby in the squares and streets*” (Service user, FGD 6)

Service-users commented that their satisfaction would be enhanced if the service (in this context, seen as synonymous with “the government”) could provide economic support, i.e. To help them to set up businesses and build their own houses:

“*The government is offering us everything except milk of the mule. We need the organization to help us make some sorts of trades if it can*” (Service user, FGD 6)

#### Content of communication

For most, although medication was seen as a pre-requisite, advice and counselling provided by healthcare workers was appreciated:

“*it is advising me that reaches my heart*” (Service user, FGD 6)

However, there was variation in what service-users and caregivers wanted to hear from healthcare workers. Some wanted minimal medication support only, one service user wanted “worrying information” withheld, whilst another service user wanted more “education” about his illness.

#### Quality of communication

How information was communicated to service-users was as important as what was conveyed. Appointments needed to be the right length and frequency. Some were concerned about the impact of longer appointments upon the work of service-users and caregivers: “*too much talk cannot be loaded on a donkey*”. Desired frequency of appointments varied, with most service users preferring more frequent appointments (e.g. once every 1–2 months) to support consistency of behaviours:

“*At least, if we do not meet them once in a month, we may forget what we are supposed to do. For example, if people are not using the road, grasses will grow on it*”.

(Service user, FGD 6)

For others, once they were feeling better, less frequent appointments (e.g. two to three times a year) were felt to be sufficient. Although at times, many service-users preferred a caregiver to be present, there were some circumstances in which service-users preferred to meet with healthcare workers alone: “*there are some issues that need to be private*”. This preference was recognised by caregivers. Meeting alone was perceived to facilitate openness, truthfulness and concentration, all of which were linked to satisfaction. One service user suggested that when they felt “better” they preferred to be alone.

#### Healthcare worker characteristics

Service users appreciated healthcare workers treating them with respect, which they linked to concepts of: warmth, tolerance, acceptance, hopefulness, calm, patience, courtesy, asking questions and listening well to service users’ responses, which were identified as the foundation of successful therapeutic relationship:

“*If a parent is rearing his/her child with courtesy and due respect, the child will be fruitful*” (Service-user, FGD 6)

Confidentiality was presumed, with service-users stating that they would be embarrassed, offended and sad if a healthcare worker disclosed their “secrets” to others. If they found out that confidentiality had been broken behind their backs, this would result in a severance of trust and prevent further disclosures. However, conversely, some were untroubled by having clinical interactions in the presence of others, feeling that other service users could learn from what was said.

#### Clinical environment

Environmental characteristics were not spontaneously discussed but when brought up by the interviewer, service-users and caregivers agreed they were an important contributor to satisfaction. It was important that the toilet was available and clean: “if the toilet is not clean, we will have another sickness” (Service-user, FGD 6). It was desired that the waiting area should be clean, shaded, quiet and separate:

“*As patients come from far, they may get tired and need to take rest till their time comes*” (Service-user, FGD 10).

### Quantitative study

In the first sample, endorsement of service satisfaction items ranged from 76.5 to 93.0% among service users and 74.9 to 99.0% among caregivers (Additional file 1). A three factor solution accounted for 48.1% of variance among service-users and 35.6% among caregivers (Additional file 2). Factors can be characterized as follows: service-users: 1. Interpersonal aspects of care; 2. Efficacy; Technical competence and facilities; caregivers: 1. Technical competency; 2. Efficacy; 3. Communication. Among service users, all but two items (“waiting time was acceptable”; “my privacy is respected”) had item-test correlations of >0.40. Average inter-item correlation ranged from 0.27 to 0.31. Overall Cronbach’s alpha was 0.89 (Table 1). Among caregivers, all but one item (“waiting time was acceptable”) had item-test correlations of >0.40. Average inter-item correlation ranged from 0.22 to 0.24. Overall Cronbach’s alpha was 0.85 (Table 1). Strength of agreement for the kappa coefficient was fair to substantial (0.21–0.66) for some items only [35] (see Table 2). After adjustment for relative wealth and sex, four out of eight adherence variables were associated with service satisfaction (remembers to take medication adjusted odds ratio (AOR) = 2.74, 95% CI = 1.27–5.91; has not stopped taking medication due to feeling worse AOR = 2.94, 95% CI = 1.37–6.32; has not felt hassled to follow treatment plan AOR = 3.39, 95% CI = 1.64–7.01; rarely forgets to take medication AOR = 0.42, 95% CI = 0.20–0.87). Among caregivers, remembers to take medication (AOR = 4.28, 95% CI = 2.22–8.26) was associated with satisfaction, in addition to the four items found to be associated among service users. Among service users, two therapeutic alliance items retained statistical significance in multivariable analyses (treatment you receive is moderately/completely right for you AOR = 4.28, 95% CI = 1.5–12.22; feel better after meeting with the healthcare worker AOR = 7.32, 95% CI = 2.29–23.40). The same items were associated with satisfaction among caregiver respondents (see Additional file 3).

**Table 2:** Sample 1. Test-retest reliability of satisfaction scale in service users (*n* = 50)

Item	Kappa (SE)
The health worker treated me with courtesy	−0.09 (0.118)
The health worker listened to me carefully	0.00
The health worker explained me things in a way I understood	0.25 (0.106)
The health facility was clean	−0.02 (0.060)
The waiting room was clean	0.21 (0.104)
The latrine was clean	0.14 (0.103)
The waiting time was acceptable	0.28 (0.130)
I have enough time to discuss with health worker	−0.03 (0.076)
I was give information in a way I understood	0.40 (0.129)
I received helpful advice	0.31 (0.103)
Administrative staff treated me with courtesy and respect	0.41 (0.109)
The health worker involved my family helpfully	0.14 (0.108)
My privacy is respected	0.40 (0.123)
I have the opportunity for follow up with the same health worker	0.34 (0.128)
My personal information is kept confidential	0.00
Referral to specialist is possible	0.49 (0.085)
The service is effective at decreasing symptoms	0.09 (0.102)
The service is effective at decreasing relapses	0.11 (0.102)
The service is effective at helping with economic problems	0.11 (0.115)
It is possible to see the health worker when needed	0.35 (0.110)

**Fig. 2:**
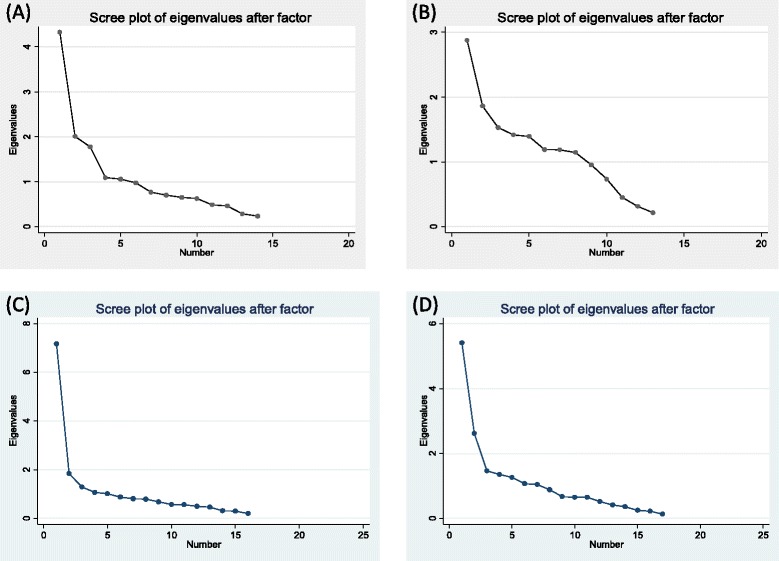
Scree plots of Eigenvalues. **a** Sample 1. Service users. **b** Sample 1. Caregivers. **c** Sample 2. Service users. **d** Sample 2. Caregivers

In our second sample, endorsement of service satisfaction items ranged from 50.0 to 98.0% among service users and 56.7 to 98.6% among caregivers (Table 3). Scree plots for service-users and caregivers in sample 2 were more unambiguously indicative of a single factor solution (Fig. 2; Table 4). Among service users, all but two items had item-test correlations of >0.40 (Table 5). Average inter-item correlation ranged from 0.30 to 0.33. Overall Cronbach’s alpha was 0.92 (Table 5). Among caregivers, all but two items had item-test correlations of >0.40. Average inter-item correlation ranged from 0.30 to 0.32. Overall Cronbach’s alpha was 0.92 (Table 5). The final scale can be found in Additional files 4 (English version) and 5 (Amharic version).

**Table 3:** Sample 2: distribution of service satisfaction

Item	Service users (*n* = 150)				Caregiver (*n* = 150)			
	Strongly disagree *N* (%)	Disagree *N* (%)	Agree *N* (%)	Strongly agree *N* (%)	Strongly disagree *N* (%)	Disagree *N* (%)	Agree *N* (%)	Strongly agree *N* (%)
The health worker treated me with courtesy	0 (0.0)	5 (3.3)	104 (69.3)	41 (27.3)	0 (0.0)	5 (3.3)	83 (55.3)	62 (41.3)
The health worker listened to me carefully	0 (0.0)	3 (2.0)	110 (73.3)	37 (24.7)	0 (0.0)	7 (4.7)	90 (60.4)	52 (34.9)
The health worker explained me things in a way I understood	1 (0.7)	5 (3.4)	115 (77.2)	28 (18.8)	0 (0.0)	5 (3.3)	112 (74.7)	33 (22.0)
The health facility was clean	0 (0.0)	5 (3.3)	103 (68.7)	42 (28.0)	1 (0.7)	1 (0.7)	88 (58.7)	60 (40.0)
The waiting area was clean	2 (1.3)	12 (8.0)	118 (78.7)	18 (12.0)	0 (0.0)	13 (8.7)	110 (73.3)	27 (18.0)
The latrine was clean [don’t use toilet SU *n* = 47 (31.3); CG *n* = 46 (30.7)]	3 (2.0)	12 (8.0)	65 (43.3)	23 (15.3)	4 (2.7)	10 (6.7)	53 (35.3)	37 (24.7)
The waiting time was acceptable	2 (1.3)	27 (18.0)	109 (72.7)	12 (8.0)	2 (1.3)	25 (16.7)	109 (72.7)	14 (9.3)
I have enough time to discuss with health worker	0 (0.0)	13 (8.7)	117 (78.0)	20 (13.3)	3 (2.0)	8 (5.3)	110 (73.3)	29 (19.3)
I was give information in a way I understood	1 (0.7)	6 (4.0)	124 (82.7)	19 (12.7)	1 (0.7)	5 (3.3)	115 (76.7)	29 (19.3)
I received helpful advice	1 (0.7)	12 (8.1)	97 (65.1)	39 (26.2)	2 (1.3)	4 (2.7)	85 (56.7)	59 (39.3)
Administrative staff treated me with courtesy and respect	2 (1.3)	3 (2.0)	124 (82.3)	21 (14.0)	1 (0.7)	8 (5.4)	111 (74.5)	29 (19.5)
The health worker involved my family helpfully	1 (0.7)	2 (1.3)	124 (82.7)	23 (15.3)	0 (0.0)	4 (2.7)	122 (81.3)	24 (16.0)
My privacy is respected	1 (0.7)	6 (4.0)	128 (85.3)	15 (10.0)	1 (0.7)	14 (9.4)	114 (76.5)	20 (13.4)
I have the opportunity for follow up with the same health worker	1 (0.7)	14 (9.3)	123 (82.0)	12 (8.0)	1 (0.7)	14 (9.4)	118 (79.2)	16 (10.7)
My personal information is kept confidential	1 (0.7)	6 (4.0)	98 (65.3)	45 (30.0)	0 (0.0)	8 (5.3)	84 (56.0)	58 (38.7)
Referral to specialist is possible	2 (1.3)	4 (2.7)	101 (67.3)	43 (28.7)	0 (0.0)	4 (2.7)	96 (64.4)	49 (32.9)
The treatment reduced my symptoms	0 (0.0)	14 (9.4)	79 (53.0)	56 (47.6)	0 (0.0)	13 (8.7)	65 (43.3)	72 (48.0)
The treatment reduced relapse of my illness	2 (1.3)	17 (11.3)	77 (51.3)	54 (36.0)	1 (0.7)	19 (12.7)	60 (40.0)	70 (46.7)
The treatment helped me to improve my income	2 (1.4)	33 (22.3)	71 (48.0)	42 (28.4)	6 (4.0)	38 (25.3)	56 (37.3)	50 (33.3)
I can get a health worker’s help any time I need	1 (0.7)	3 (2.0)	123 (82.0)	23 (15.3)	0 (0.0)	8 (5.3)	113 (75.3)	29 (19.3)
It was easy to come to the hospital	5 (3.3)	22 (14.7)	113 (75.3)	10 (6.7)	6 (4.0)	19 (12.7)	107 (71.3)	18 (12.0)
I had enough time to come to the hospital	2 (1.3)	36 (24.0)	103 (68.7)	9 (6.0)	1 (0.7)	27 (18.0)	112 (74.7)	10 (6.7)
I had enough money to come and get treatment	12 (8.0)	63 (42.0)	72 (48.0)	3 (2.0)	7 (4.7)	58 (38.7)	79 (52.7)	6 (4.0)
I would advise my family to come to the hospital	2 (1.3)	7 (4.7)	89 (59.3)	52 (34.7)	4 (2.7)	9 (6.0)	69 (46.0)	68 (45.3)

**Table 4:** Sample 2- Loadings for a single factor solution among service-users and caregivers

	Service-users	Care-givers
% of variance explained by model	38.9	28.5
Item	Factor 1	Factor 1
The health worker treated me with courtesy	0.58	0.61
The health worker listened to me carefully	0.45	0.62
The health worker explained me things in a way I understood	0.51	0.49
The health facility was clean	0.60	0.77
The waiting area was clean		0.45
The latrine was clean	0.38	0.37
The waiting time was acceptable	0.35	0.35
I have enough time to discuss with health worker	0.44	0.53
I was give information in a way I understood	0.47	0.48
I received helpful advice	0.70	0.66
Administrative staff treated me with courtesy and respect	0.57	0.44
The health worker involved my family helpfully	0.57	0.47
My privacy is respected	0.44	0.55
I have the opportunity for follow up with the same health worker	0.33	0.44
My personal information is kept confidential	0.78	0.74
Referral to specialist is possible	0.83	0.70
The treatment reduced my symptoms	0.68	0.69
The treatment reduced relapse of my illness	0.80	0.79
The treatment helped me to improve my income	0.74	0.70
I can get a health worker’s help any time I need	0.59	0.52
It was easy to come to the hospital	0.41	0.50
I had enough time to come to the hospital	0.53	0.47
I had enough money to come and get treatment	0.36	0.36
I would advise my family to come to the hospital	0.85	0.72

**Table 5:** Sample 2: Internal consistency and item-item correlations

	Service Users		Caregivers	
Item	Item-test correlation	Average item-item correlation	Item-test correlation	Average item-item correlation
The health worker treated me with courtesy	0.59	0.31	0.62	0.31
The health worker listened to me carefully	0.50	0.32	0.66	0.31
The health worker explained me things in a way I understood	0.59	0.31	0.54	0.32
The health facility was clean	0.59	0.31	0.76	0.30
The waiting area was clean	0.32	0.31	0.51	0.32
The latrine was clean	0.41	0.31	0.39	0.32
The waiting time was acceptable	0.46	0.33	0.43	0.32
I have enough time to discuss with health worker	0.55	0.32	0.58	0.31
I was give information in a way I understood	0.58	0.31	0.54	0.32
I received helpful advice	0.71	0.31	0.71	0.31
Administrative staff treated me with courtesy and respect	0.62	0.31	0.52	0.32
The health worker involved my family helpfully	0.66	0.31	0.56	0.31
My privacy is respected	0.54	0.32	0.62	0.31
I have the opportunity for follow up with the same health worker	0.41	0.32	0.50	0.32
My personal information is kept confidential	0.75	0.31	0.72	0.31
Referral to specialist is possible	0.78	0.30	0.67	0.31
The treatment reduced my symptoms	0.64	0.31	0.66	0.31
The treatment reduced relapse of my illness	0.75	0.30	0.75	0.30
The treatment helped me to improve my income	0.69	0.31	0.64	0.30
I can get a health worker’s help any time I need	0.66	0.31	0.54	0.31
It was easy to come to the hospital	0.49	0.32	0.54	0.32
I had enough time to come to the hospital	0.62	0.31	0.52	0.32
I had enough money to come and get treatment	0.38	0.33	0.37	0.32
I would advise my family to come to the hospital	0.78	0.30	0.68	0.31

Service user overall Cronbach’s alpha = 0.92; Caregiver overall Cronbach’s alpha = 0.92

## Discussion

Our findings suggest that we have developed a scale that is a useful starting point for capturing patients’ subjective responses to the experience of using services. Results suggest that revisions made to version 0.0 of the Mental Health Service Satisfaction Scale (MHSSS) in response to study 1 findings and expert review were successful in leading to an improved instrument, with excellent internal consistency and factor loadings more clearly indicative of a unidimensional construct. Results from the qualitative study were useful in interpreting satisfaction scores, triangulating observed associations: contributing to understanding how experiences are related to satisfaction among accessing services for SMD in rural Ethiopia. Factors identified in analysis of the first quantitative sample coincide with core concepts of service satisfaction as reported in the literature and reflected key themes which emerged from our qualitative study: interpersonal factors, efficacy, communication, technical competency and adequacy of facilities. Consistent with findings from our previous qualitative study, quantitative and qualitative results were generally indicative of consensus between service-users and caregivers regarding the main dimensions of service satisfaction [36]. However, as before, there was some evidence of differences in prioritisation. In our previous study, caregivers identified high quality medication management as the most important aspect of a service for people with SMD whereas users valued the supportive nature of care received. In the current study, factor structure suggested that whilst caregivers felt that technical competency was the most important component of satisfaction, interpersonal aspects of care were the key priority for service users.

Consistent with our previous research, observed impact of pharmacological therapy on service-user’s lives was described as being the most important component of satisfaction with the service [36]. When we examined convergent validity, the largest odds ratios for associations with satisfaction were for items measuring aspects of therapeutic alliance related to impact (“how much do you feel the treatment you are receiving is right for you?”, “how do you feel after a meeting with the healthcare worker?”). In fact, there is a lack of research examining the links between satisfaction and objective outcome measures [1]. However, this perceived link between satisfaction and efficacy is potentially important. In other settings, perceptions of quality have predicted utilization [37].

Apart from efficacy, the emphasis placed on interpersonal aspects of care: content and quality of communication, personal characteristics of the healthcare worker are consistent with findings elsewhere which suggest that interpersonal aspects of care account for a greater degree of variance in satisfaction as compared to technical aspects [1]. Qualitative studies carried out in the West highlight the value placed by service users upon healthcare workers knowing them beyond their symptoms of mental illness [12, 38, 39]. Flexibility of approach, listening and responding to an individual’s situation is perceived as showing respect [12]. Experiencing a connection with others and the promotion of hope and empowerment are core recovery processes [40].

Findings suggest that female service-users and those who report being less wealthy are less satisfied with the service they receive. Further research is necessary to understand possible reasons for this. Our qualitative research was not designed in such a way to enable comparison of responses by sociodemographic characteristics: we had an insufficient number of in-depth interviews and contributions to focus group discussions were not analysed by sociodemographic characteristics. However, as new services continue to be rolled out, it will be important to consider the implications for equity.

The consistency of our results with existing models of service satisfaction is reassuring. However, when considering the implications of findings, it is important to take into account both longstanding criticisms of measures of satisfaction, as well as particular limitations inherent to our study design.

Consistently high levels of service satisfaction captured in studies have prompted concerns about the interpretability and meaning of measures [12]. The power imbalance between users and health providers is commonly heightened in mental health care [41], whilst social marginalisation and exclusion remain a common part of the experience of living with severe mental illness [42, 43]. Capturing service satisfaction/dissatisfaction in low income settings where there have been no biomedical services for mental disorder is an additional measurement challenge. In previous studies from Ethiopia, the lack of previous experience of care limited participants’ abilities to critically reflect on the potential advantages and disadvantages of the new service model [19, 20], whilst the restricted nature of the social roles of people living with severe mental illness may inhibit expression of opinion [36]. Therefore, perhaps particularly in settings where there was previously no service at all, it is important not to elide satisfaction with quality of care: satisfaction might merely be a reflection of having access to a service and (low) expectations being met. It is a limitation of our study design that service-users were not included as part of the expert panel which reviewed version 0.0 of the instrument.

Findings from research carried out in the US suggest that quality and satisfaction instruments which refer to a specific encounter offer more accurate representation of the quality of care received [44]. Although our instrument asked participants to answer with reference to their most recent clinic encounter, in practice, the extent to which participants were able to isolate their most recent interaction from their long-term experience of using the service is unclear. In this setting, where, effectively, the Butajira clinic is the only biomedical service available for SMD, this was perhaps marginally less problematic than a US setting where service-users may confuse care received from several different services.

With the exception of a few items, test-retest reliability results were lower than expected, with kappa’s indicating agreement no better than that expected by chance. The extent to which we would expect consistency across time among symptomatic patients in this setting is unclear. In addition, the high prevalence of positive ratings means that chance agreement is also high and therefore kappa is reduced accordingly [45]. Unfortunately, resources did not allow us to carry out test-retest reliability testing for sub-study 2.

## Conclusions

As new mental health services are rolled out in low and middle income country settings, if improved patient outcomes are to be achieved, it will be essential to move beyond the goal of achieving coverage towards a dual focus upon ensuring delivery of quality of care [21]. Findings from our mixed methods study demonstrate important linkages between satisfaction with services and established dimensions of quality: adherence, therapeutic alliance, efficacy, positive perceptions of communication, technical competency and healthcare worker characteristics. Results are suggestive of concurrence between the construct of satisfaction of users of a service for SMD in rural Ethiopia with the model of satisfaction described in the quality of care literature (see Fig. 1). Our findings indicate that a measure of satisfaction with services, such as our MHSSS, might be a useful candidate for inclusion into the new toolkit of measures needed to facilitate monitoring of service satisfaction and tracking over time which are the foundations of performance improvement in healthcare. Further research will be necessary to identify a set of appropriate indicators of quality of mental healthcare in LMIC [21]: potential domains for consideration might include: continuity of care, care co-ordination, treatment, patient outcomes. Quality audits by experts might also be utilized as part of continuous improvement cycles, Care should be taken to involve service users in this process [13]. There is no doubt that measuring satisfaction alone is insufficient to capture quality of care.

It would be informative to investigate how the final MHSSS performs in other independent samples and other comparable settings, for example, among service users in other rural sub-Saharan African countries. Confirmatory factor analysis could be applied to examine the cross-setting applicability of the single dimension. Use of item response theory approaches (e.g. Rasch analysis) might also be considered in combination with factor analysis, with a view to item reduction to enhance the clinical utility of the instrument. The analysis presented here provides a framework for hypothesis generation regarding the impact of removal of poorly performing items.

Although our research findings suggest that the factors that determine satisfaction with mental health services may be broadly similar across cultures, universality of constructs related to quality cannot be assumed: without undertaking similar exploratory work, cultural differences cannot be ruled out. It will be necessary to carry out further formative research involving service users, providers and policymakers in order to operationalise quality of care of task-shared services in different cultural settings. Successful implementation of high quality care will be contingent upon broader health system developments, including the development of effective mental health information systems, which are essential to delivering measurement-based care but currently largely absent from healthcare systems in low income countries [46].

## Additional files

Additional file 1: Frequency distribution of responses to items on Mental Health Service Satisfaction scale. (DOCX 13 kb)
Additional file 2: Exploratory factor analysis of responses to the initial Mental Health Service Satisfaction scale in sample 1. (DOCX 14 kb)
Additional file 3: Univariate and multivariable analysis of association between medication adherence, therapeutic alliance and service satisfaction. (DOCX 17 kb)
Additional file 4: Mental health service satisfaction scale (English version). Final English version of the mental health service satisfaction scale. (DOCX 16 kb)
Additional file 5: Mental health service satisfaction scale (Amharic version). Final Amharic version of the mental health service satisfaction scale. (PDF 309 kb)

## Abbreviations

MHSSS: Mental health service satisfaction scale
O-PAHC: Out-patient: patient assessment of healthcare
SMD: Severe mental disorder
TaSCS: Task-sharing
VSSS: Verona service satisfaction scale
