# Supplementary material for: Developing a measure of mental health service satisfaction for use in low income countries: a mixed methods study
Source: BMC Health Serv Res. 2017 Mar 9;17:183. doi: 10.1186/s12913-017-2126-2 (PMC5343366; doi:10.1186/s12913-017-2126-2)
Supplement: Additional file 1: — Frequency distribution of responses to items on Mental Health Service Satisfaction scale. (DOCX 13 kb) [file 12913_2017_2126_MOESM1_ESM.docx]

**Additional File 1. Sample 1: Frequency distribution of responses to items on Mental Health Service Satisfaction scale**

| **Item** | **Service users (n=200)** | | | | **Caregiver (n=200)** | | | |
| --- | --- | --- | --- | --- | --- | --- | --- | --- |
|  | **Strongly disagree**  **N (%)** | **Disagree**  **N (%)** | **Agree**  **N (%)** | **Strongly agree**  **N (%)** | **Strongly disagree**  **N (%)** | **Disagree**  **N (%)** | **Agree**  **N (%)** | **Strongly agree**  **N (%)** |
| The health worker treated me with courtesy | 0 (0.0) | 7 (3.5) | 180 (90.5) | 12 (6.0) | 0 (0.0) | 2 (1.0) | 183 (92.0) | 14 (7.0) |
| The health worker listened to me carefully | 2 (1.0) | 9 (4.7) | 176 (91.7) | 5 (2.6) | 0 (0.0) | 6 (3.2) | 176 (92.6) | 8 (4.2) |
| The health worker explained me things in a way I understood | 0 (0.0) | 14 (7.1) | 179 (90.4) | 5 (2.5) | 0 (0.0) | 3 (1.5) | 190 (95.5) | 6 (3.0) |
| The health facility was clean | 0 (0.0) | 6 (3.0) | 186 (93.9) | 6 (3.0) | 0 (0.0) | 7 (3.5) | 186 (93.9) | 5 (2.5) |
| The latrine was clean | 0 (0.0) | 20 (10.2) | 174 (88.3) | 3 (1.5) | 1 (0.5) | 16 (8.0) | 180 (90.0) | 3 (1.5) |
| The waiting time was acceptable | 1 (0.5) | 33 (16.7) | 158 (79.8) | 6 (3.0) | 0 (0.0) | 28 (14.1) | 167 (84.3) | 3 (1.5) |
| I have enough time to discuss with health worker | 1 (0.5) | 23 (11.6) | 172 (86.4) | 3 (1.5) | 1 (0.5) | 18 (9.0) | 177 (88.9) | 3 (1.5) |
| I was give information in a way I understood | 1 (0.5) | 17 (8.5) | 179 (89.5) | 3 (1.5) | 0 (0.0) | 12 (6.0) | 185 (92.5) | 3 (1.5) |
| I received helpful advice | 1 (0.5) | 26 (13.2) | 165 (83.8) | 5 (2.5) | 0 (0.0) | 15 (7.5) | 177 (88.9) | 7 (3.5) |
| Administrative staff treated me with courtesy and respect | 1 (0.5) | 14 (7.0) | 177 (88.8) | 8 (4.0) | 1 (0.5) | 13 (6.5) | 178 (89.0) | 8 (4.0) |
| The health worker involved my family helpfully | 2 (1.0) | 8 (4.0) | 181 (91.0) | 8 (4.0) | 0 (0.0) | 5 (2.5) | 193 (96.5) | 2 (1.0) |
| My privacy is respected | 3 (1.5) | 27 (13.7) | 165 (83.8) | 2 (1.0) | 2 (1.0) | 28 (14.1) | 166 (83.8) | 2 (1.0) |
| I have the opportunity for follow up with the same health worker | 0 (0.0) | 30 (15.0) | 168 (84.0) | 2 (1.0) | 1 (0.5) | 30 (15.0) | 168 (84.0) | 1 (0.5) |
| My personal information is kept confidential | 2 (1.0) | 13 (6.5) | 182 (91.5) | 2 (1.0) | 0 (0.0) | 18 (9.0) | 180 (90.0) | 2 (1.0) |
| Referral to specialist is possible | 2 (1.0) | 14 (7.0) | 183 (91.5) | 1 (0.5) | 0 (0.0) | 8 (4.0) | 189 (95.0) | 2 (1.0) |
| The service is effective at decreasing symptoms | 3 (1.5) | 18 (9.0) | 165 (82.9) | 13 (6.5) | 2 (1.0) | 7 (3.5) | 170 (85.9) | 19 (9.6) |
| The service is effective at decreasing relapses | 2 (1.0) | 20 (10.0) | 163 (81.5) | 15 (7.5) | 1 (0.5) | 15 (7.6) | 165 (83.3) | 17 (8.6) |
| The service is effective at helping with economic problems | 2 (1.0) | 45 (22.5) | 146 (73.0) | 7 (3.5) | 1 (0.5) | 49 (24.6) | 144 (72.4) | 5 (2.5) |
| It is possible to see the health worker when needed | 0 (0.0) | 18 (9.4) | 170 (88.5) | 4 (2.1) | 2 (1.0) | 16 (8.1) | 176(88.9) | 4 (2.0) |
