# Supplementary material for: Developing a measure of mental health service satisfaction for use in low income countries: a mixed methods study
Source: BMC Health Serv Res. 2017 Mar 9;17:183. doi: 10.1186/s12913-017-2126-2 (PMC5343366; doi:10.1186/s12913-017-2126-2)
Supplement: Additional file 2: — Exploratory factor analysis of responses to the initial Mental Health Service Satisfaction scale in sample 1. (DOCX 14 kb) [file 12913_2017_2126_MOESM2_ESM.docx]

**Additional File 2. Sample 1: Loadings for 1 to 3 factor solutions among service-users and caregivers**

|  | **Service-users** | **Care-givers** | **Service-users** | | **Caregivers** | | **Service-users** | | | **Caregivers** | | |
| --- | --- | --- | --- | --- | --- | --- | --- | --- | --- | --- | --- | --- |
| **% of variance explained by model** | **28.1** | **18.8** | **41.1** | | **28.1** | | **48.1** | | | **35.6** | | |
| **Item** | **Factor 1** | **Factor 1** | **Factor 1** | **Factor 2** | **Factor 1** | **Factor 2** | **Factor 1** | **Factor 2** | **Factor 3** | **Factor 1** | **Factor 2** | **Factor 3** |
| The health worker treated me with courtesy | 0.53 | 0.43 | 0.43 |  |  | 0.34 | 0.46 |  |  |  |  | 0.99 |
| The health worker listened to me carefully | 0.68 | 0.51 | 0.61 |  | 0.34 | 0.37 | 0.73 |  |  |  |  | 0.64 |
| The health worker explained me things in a way I understood | 0.72 | 0.56 | 0.67 |  | 0.36 | 0.44 | 0.66 |  |  |  | 0.36 | 0.42 |
| The health facility was clean |  | 0.49 |  |  | 0.49 |  |  |  | 0.44 | 0.46 |  |  |
| The waiting room was clean | 0.37 | 0.48 | 0.34 |  | 0.58 |  |  |  | 0.51 | 0.59 |  |  |
| The latrine was clean |  | 0.52 |  |  | 0.59 |  |  |  | 0.39 | 0.66 |  |  |
| The waiting time was acceptable |  |  |  |  | 0.36 |  |  |  | 0.47 | 0.34 |  |  |
| I have enough time to discuss with health worker | 0.54 | 0.60 | 0.54 |  | 0.64 |  | 0.35 |  | 0.67 | 0.62 |  |  |
| I was given information in a way I understood | 0.87 | 0.69 | 0.86 | 0.31 | 0.61 |  | 0.77 |  | 0.37 | 0.60 |  |  |
| I received helpful advice | 0.73 | 0.49 | 0.63 | 0.35 | 0.42 |  | 0.61 | 0.34 |  | 0.36 |  | 0.30 |
| Administrative staff treated me with courtesy and respect | 0.42 | 0.45 | 0.34 |  | 0.51 |  | 0.34 |  |  | 0.48 |  |  |
| The health worker involved my family helpfully | 0.69 | 0.57 | 0.57 | 0.40 | 0.52 |  | 0.56 | 0.39 |  | 0.48 |  |  |
| My privacy is respected | 0.34 | 0.33 | 0.35 |  |  |  | 0.33 |  |  |  |  |  |
| I have the opportunity for follow up with the same health worker | 0.55 | 0.33 | 0.60 |  |  |  | 0.50 |  | 0.38 | 0.31 |  |  |
| My personal information is kept confidential | 0.40 |  | 0.41 |  |  |  | 0.47 |  |  | 0.31 |  |  |
| Referral to specialist is possible | 0.48 |  | 0.48 |  |  |  | 0.60 |  |  |  |  |  |
| The service is effective at decreasing symptoms | 0.56 | 0.49 |  | 0.81 |  | 0.95 |  | 0.81 |  |  | 0.90 |  |
| The service is effective at decreasing relapses | 0.64 | 0.47 |  | 0.96 |  | 0.80 |  | 0.96 |  |  | 0.82 |  |
| The service is effective at helping with economic problems | 0.60 | 0.43 | 0.33 | 0.61 |  | 0.35 | 0.31 | 0.61 |  | 0.33 | 0.39 |  |
| It is possible to see the health worker when needed | 0.66 | 0.51 | 0.62 |  | 0.56 |  | 0.62 |  |  | 0.56 |  |  |
