# Supplementary material for: Developing a measure of mental health service satisfaction for use in low income countries: a mixed methods study
Source: BMC Health Serv Res. 2017 Mar 9;17:183. doi: 10.1186/s12913-017-2126-2 (PMC5343366; doi:10.1186/s12913-017-2126-2)
Supplement: Additional file 3: — Univariate and multivariable analysis of association between medication adherence, therapeutic alliance and service satisfaction. (DOCX 17 kb) [file 12913_2017_2126_MOESM3_ESM.docx]

**Additional File 3. Sociodemographic factors, indicators of adherence and therapeutic alliance: associations with service satisfaction**

| Item | Service users | | | Caregivers | | |
| --- | --- | --- | --- | --- | --- | --- |
|  | Mean satisfaction=2.92 | | | Mean satisfaction= 2.94 | | |
|  | Prevalence of service satisfaction (>mean) | Associations with service satisfaction ( >mean) | | Prevalence of service satisfaction (>mean) | Associations with service satisfaction (>mean) | |
|  |  | OR (95% CI) | *AOR (95% CI) |  | OR (95% CI) | **AOR (95% CI) |
|  |  |  |  | 140 (70.0) |  |  |
| **Sociodemographic** |  |  |  |  |  |  |
| Sex |  |  |  |  |  |  |
| Male | 92(72.4) |  |  | 107 (67.7) |  |  |
| Female | 42 (57.5) | 0.52 (0.28-0.95) |  | 33 (78.6) | 1.75 (0.77-3.95) |  |
| Age (years) |  |  |  |  |  |  |
| <25 | 40 (62.5) |  |  | 22 (62.9 | --------------------- |  |
| 25-49 | 81 (70.4) | 1.43 (0.75-2.74) |  | 83 (70.3) | 1.40 (0.63-3.11) |  |
| >50 | 13 (65.0) | 1.11 (0.39-3.2) |  | 33 (73.3) | 1.63 (0.62-4.26) |  |
| Education |  |  |  |  |  |  |
| Can read and write | 97 (69.8) | 1.54 (0.82-2.91) |  | 12 (71.3) | 1.21 (0.61-2.40) |  |
| Relative wealth |  |  |  |  |  |  |
| Less | 54 (51.4) |  |  | 55 (59.8) |  |  |
| Equal | 78 (85.7) | 5.67 (2.66-12.05) |  | 83 (78.3) | 2.43 (1.28-4.59) |  |
| Greater | 2 (50.0) | 0.94 (0.13-7.02) |  | 1 (100.0) | --------------------- |  |
| Diagnosis |  |  |  |  |  |  |
| Schizophrenia | 70(64.8) |  |  | 65 (68.4) |  |  |
| Bipolar disorder | 38 (67.9) | 1.15 (0.56-2.28) |  | 33 (66.0) | 0.90 (0.43-1.86) |  |
| Major depressive disorder | 26 (74.3) | 1.57 (0.66-3.71) |  | 25 (75.88) | 1.44 (0.58-3.59) |  |
| **Adherence** |  |  |  |  |  |  |
| Remembers to take medication | 113 (72.4) | 2.88 (1.42-5.83) | 2.74 (1.27-5.91) | 41 (51.9) | 4.04 (2.06-7.95) | 4.28 (2.22-8.26) |
| Has not missed doses in last 2 weeks | 94(72.9) | 2.08 (1.12-3.87) | 1.92 (0.98-3.77) | 87 (73.7) | 1.70 (0.91-3.16) |  |
| Has not stopped medication, feeling worse | 107 (71.3) | 2.60 (1.29-5.23) | 2.94 (1.37-6.32 | 89 (74.8) | 2.03 (1.06-3.86) | 2.02 (1.06-3.87) |
| Has not forgotten to take medication due to travelling | 102 (68.9) | 1.77(0.89-3.54) |  | 101 (79.5) | 3.88 (1.97-7.67) | 3.83 (1.98-7.41) |
| Took medication yesterday | 55 (64.0) | 0.81 (0.44-1.46) |  | 70 (71.4) | 1.23 (0.67-2.27) |  |
| Has not stopped medication, feeling better | 100 (70.4) | 1.71(0.89-3.28) |  | 96 (72.7) | 1.60 (0.84-3.03) |  |
| Has not felt hassled to follow treatment plan daily | 107 (74.3) | 3.11(1.60-6.05) | 3.39 (1.64-7.01) | 97 (79.5) | 3.40 (1.76-6.56) | 3.29 (1.73-6.26) |
| Frequency of forgetting to take medication |  |  |  |  |  |  |
| Never | 97(74.197.7) |  |  | 127 (72.6) |  |  |
| Rarely/sometimes | 29 (53.7) | 0.41 (0.21-0.80) | 0.42 (0.20-0.87) | 9 (45.0) | 0.31 (0.12-0.81) | 0.24 (0.09-0.66) |
| A lot of the time/ almost all the time | 7(50.0) | 0.35 (0.11-1.09) | 0.31 (0.09-1.11) | 1 (50.0) | 0.38 (0.02-6.24) | 0.23 (0.01-3.81) |
| **Therapeutic alliance** |  |  |  |  |  |  |
| How much do you feel understood by the HCW? |  |  |  |  |  |  |
| Moderately/completely | 124(68.9) | 2.21 (0.86-5.67) |  | 137 (70.3) | 0.79 (0.08-7.77) |  |
| Do you feel criticised/blamed by the HCW? |  |  |  |  |  |  |
| Not at all/a little | 117 (66.5) | 0.87 (0.34-2.23) |  | 121 (68.8) | 0.61 (0.21-1.74) |  |
| How much do you feel the HCW is concerned/cares? |  |  |  |  |  |  |
| Moderately/completely | 127 (70.2) | 3.92 (1.34-11.58) | 1.46 (0.92-2.32) | 135 (71.1) | 2.45 (0.68-8.90) |  |
| How much do you feel the treatment you are currently receiving is right for you? |  |  |  |  |  |  |
| Moderately/completely | 128 (71.9) | 8.19 (2.69-24.98) | 4.28 (1.50-12.22) | 138 (71.9) | 7.67 (1.44-40.68) | 7.10 (1.35-37.36) |
| How do you feel after a meeting with the HCW? |  |  |  |  |  |  |
| Unchanged/worse | 6 (27.3) |  |  | 3 (17.7) |  |  |
| Better | 128 (71.9) | 6.83 (2.41-19.73) | 7.32 (2.29-23.40) | 137 (74.9) | 13.90 (3.49-55.27) | 12.08 (3.27-44.54) |
| *Adjusted for relative wealth, sex  **Adjusted for relative wealth | | | | | | |
