# Supplementary material for: Developing a measure of mental health service satisfaction for use in low income countries: a mixed methods study
Source: BMC Health Serv Res. 2017 Mar 9;17:183. doi: 10.1186/s12913-017-2126-2 (PMC5343366; doi:10.1186/s12913-017-2126-2)
Supplement: Additional file 4: — Mental health service satisfaction scale (English version). Final English version of the mental health service satisfaction scale. (DOCX 16 kb) [file 12913_2017_2126_MOESM4_ESM.docx]

## Mental health service satisfaction scale

Thinking back to the last appointment you had at a health facility for mental health care, please tell me how much you disagree or agree with the following statements:

| 1 | The health worker treated me with courtesy | Strongly disagree |
| --- | --- | --- |
|  |  | Disagree |
|  |  | Agree |
|  |  | Strongly agree |
| 2 | The health worker listened to me carefully | Strongly disagree |
|  |  | Disagree |
|  |  | Agree |
|  |  | Strongly agree |
| 3 | The health worker explained things to me in a way I understood | Strongly disagree |
|  |  | Disagree |
|  |  | Agree |
|  |  | Strongly agree |
| 4 | The health facility was clean | Strongly disagree |
|  |  | Disagree |
|  |  | Agree |
|  |  | Strongly agree |
| 5 | The waiting room was clean | Strongly disagree |
|  |  | Disagree |
|  |  | Agree |
|  |  | Strongly agree |
| 6 | The latrine was clean | Strongly disagree |
|  |  | Disagree |
|  |  | Agree |
|  |  | Strongly agree |
| 7 | The waiting time was acceptable | Strongly disagree |
|  |  | Disagree |
|  |  | Agree |
|  |  | Strongly agree |
| 8 | I had enough time to discuss with health worker | Strongly disagree |
|  |  | Disagree |
|  |  | Agree |
|  |  | Strongly agree |
| 9 | I was given information in a way I understood | Strongly disagree |
|  |  | Disagree |
|  |  | Agree |
|  |  | Strongly agree |
| 10 | I received helpful advice | Strongly disagree |
|  |  | Disagree |
|  |  | Agree |
|  |  | Strongly agree |
| 11 | The administrative staff treated me with courtesy and respect | Strongly disagree |
|  |  | Disagree |
|  |  | Agree |
|  |  | Strongly agree |
| 12 | The health worker involved my family helpfully | Strongly disagree |
|  |  | Disagree |
|  |  | Agree |
|  |  | Strongly agree |

## Mental health service satisfaction scale

| 13 | My privacy was respected | Strongly disagree |
| --- | --- | --- |
|  |  | Disagree |
|  |  | Agree |
|  |  | Strongly agree |
| 14 | I have the opportunity for follow up with the same health worker | Strongly disagree |
|  |  | Disagree |
|  |  | Agree |
|  |  | Strongly agree |
| 15 | My personal information is kept confidential | Strongly disagree |
|  |  | Disagree |
|  |  | Agree |
|  |  | Strongly agree |
| 16 | Referral to specialist is possible | Strongly disagree |
|  |  | Disagree |
|  |  | Agree |
|  |  | Strongly agree |
| 17 | The service is effective at decreasing symptoms | Strongly disagree |
|  |  | Disagree |
|  |  | Agree |
|  |  | Strongly agree |
| 18 | The service is effective at decreasing relapses | Strongly disagree |
|  |  | Disagree |
|  |  | Agree |
|  |  | Strongly agree |
| 19 | The service is effective at helping with economic problems | Strongly disagree |
|  |  | Disagree |
|  |  | Agree |
|  |  | Strongly agree |
| 20 | It is possible to see the health worker when needed | Strongly disagree |
|  |  | Disagree |
|  |  | Agree |
|  |  | Strongly agree |
| 21 | It was easy to attend the health facility | Strongly disagree |
|  |  | Disagree |
|  |  | Agree |
|  |  | Strongly agree |
| 22 | I had enough time to attend the health facility | Strongly disagree |
|  |  | Disagree |
|  |  | Agree |
|  |  | Strongly agree |
| 23 | I could afford to attend the health facility for treatment | Strongly disagree |
|  |  | Disagree |
|  |  | Agree |
|  |  | Strongly agree |
| 24 | I would advise my family to come to this facility for treatment if they had the same problem | Strongly disagree |
|  |  | Disagree |
|  |  | Agree |
|  |  | Strongly agree |
