# Supplementary material for: Developing a measure of mental health service satisfaction for use in low income countries: a mixed methods study
Source: BMC Health Serv Res. 2017 Mar 9;17:183. doi: 10.1186/s12913-017-2126-2 (PMC5343366; doi:10.1186/s12913-017-2126-2)
Supplement: Additional file 5: — Mental health service satisfaction scale (Amharic version). Final Amharic version of the mental health service satisfaction scale. (PDF 309 kb) [file 12913_2017_2126_MOESM5_ESM.pdf]

MENTAL HEALTH SERVICE SATISFACTION SCALE

ከዚህ ቀጥሎ የምጠይቅዎ በባለፈው ቀጠሮዎ ለህክምና በመጡ ጊዜ ከሃኪምዎ ጋር ስለነበረዎት ግንኙነትና ባገኙት የአእምሮ ሕክምና አገልግሎት ስለተሰማዎት የእርካታ መጠን በተመለከተ ነው፡፡ ስለዚህ እያንዳንዱን ጥያቄ እያነበብሁ ስጠይቅዎ ምን ያል እንደሚስማሙ ወይም እንደማይስማሙ እንዲነግሩኝ እጠይቅዎታለሁ፡፡

1	ባለፈው ለህክምና በመጣሁ ጊዜ ሐኪሙ በትህትናና በአክብሮት አስተናግደውኛል፡፡	በፍጹም አልስማማም
		አልስማማም
		እስማማለሁ
		በጣም እስማማለሁ
2	ባለፈው ለህክምና በመጣሁ ጊዜ ሐኪሙ በጥንቃቄ አዳምጠውኛል፡፡	በፍጹም አልስማማም
		አልስማማም
		እስማማለሁ
		በጣም እስማማለሁ
3	ባለፈው ለህክምና በመጣሁ ጊዜ ሐኪሙ እኔ ልረዳው በምችለው መንገድ ስለጤናየና ሕክምናየ ሁኔታ አብራርተውልኛል፡፡	በፍጹም አልስማማም
		አልስማማም
		እስማማለሁ
		በጣም እስማማለሁ
4	ባለፈው ለህክምና በመጣሁ ጊዜ በአጠቃላይ ሆስፒታሉ/ጤና ጣቢያው ንጹህ ነበር፡፡	በፍጹም አልስማማም
		አልስማማም
		እስማማለሁ
		በጣም እስማማለሁ
5	ባለፈው ለህክምና በመጣሁ ጊዜ የወረፋ መጠበቂያው አካባቢ ምቹ ነበር፡፡	በፍጹም አልስማማም
		አልስማማም
		እስማማለሁ
		በጣም እስማማለሁ
6	ባለፈው ለህክምና በመጣሁ ጊዜ ሽንት ቤቶቹ ንጹህ ነበሩ፡፡	በፍጹም አልስማማም
		አልስማማም
		እስማማለሁ
		በጣም እስማማለሁ
7	ባለፈው ለህክምና በመጣሁ ጊዜ ሐኪሙ ዘንድ ከመቅረቤ በፊት የጠበቅሁት ለረዥም ጊዜ አልነበረም፡፡	በፍጹም አልስማማም
		አልስማማም
		እስማማለሁ
		በጣም እስማማለሁ
8	ባለፈው ለህክምና በመጣሁ ጊዜ ከሐኪሙ ጋር ስለ ችግሬ ለመወያዬት በቂ ጊዜ ነበረኝ፡፡	በፍጹም አልስማማም
		አልስማማም
		እስማማለሁ
		በጣም እስማማለሁ
9	ባለፈው ለህክምና በመጣሁ ጊዜ ስለ ጤናዬ ሁኔታ ልረዳው በምችለው መንገድ መረጃ ተሰጥቶኛል፡፡	በፍጹም አልስማማም
		አልስማማም
		እስማማለሁ
		በጣም እስማማለሁ
10	ባለፈው ለህክምና በመጣሁ ጊዜ ጠቃሚ ወይም ገንቢ ምክር አግኝቻለሁ፡፡	በፍጹም አልስማማም
		አልስማማም
		እስማማለሁ
		በጣም እስማማለሁ
11	ባለፈው ለህክምና በመጣሁ ጊዜ የመድሃኒት ቤት፣የካርድ ክፍል፣የጥበቃ፣ወዘተ. ሰራተኞች በትህትናና በአክብሮት አስተናግደውኛል፡፡	በፍጹም አልስማማም
		አልስማማም
		እስማማለሁ
		በጣም እስማማለሁ
12	ባለፈው ለህክምና በመጣሁ ጊዜ ሐኪሙ ቤተሰቤን ለኔ ጠቃሚ በሆነ መንገድ እንዲሳተፉ አድርገዋል፡፡	በፍጹም አልስማማም
		አልስማማም
		እስማማለሁ
		በጣም እስማማለሁ

Mental health service satisfaction scale (continued)

13	በዚህ ሆስፒታል/ጤና ጣቢያ ከሐኪሙ ጋር በግሌ (ሌላ ሰው ሳይኖር) መነጋገር እችላለሁ፡፡	በፍጹም አልስማማም
		አልስማማም
		እስማማለሁ
		በጣም እስማማለሁ
14	በዚህ ሆስፒታል/ጤና ጣቢያ የክትትል ቀጠሮዬን ከአንድ ሐኪም ወይም ከማውቀው ባለሙያ ጋር ብቻ የመከታተል እድል አለኝ፡፡	በፍጹም አልስማማም
		አልስማማም
		እስማማለሁ
		በጣም እስማማለሁ
15	በዚህ ሆስፒታል/ጤና ጣቢያ ሐኪሙም ሆኑ ሌሎች ባለሙያዎች ግላዊ መረጃዬን በምስጢር ይይዛሉ፡፡	በፍጹም አልስማማም
		አልስማማም
		እስማማለሁ
		በጣም እስማማለሁ
16	ከዚህ ሆስፒታል/ጤና ጣቢያ አስፈላጊ ሲሆን ወደ እስፔሻሊስት መላክ ይቻላል፡፡	በፍጹም አልስማማም
		አልስማማም
		እስማማለሁ
		በጣም እስማማለሁ
17	በዚህ ሆስፒታል/ጤና ጣቢያ የማገኘው ህክምና የአእምሮ ህመም ምልክቶቼን በደንብ ያስታግስልኛል፡፡	በፍጹም አልስማማም
		አልስማማም
		እስማማለሁ
		በጣም እስማማለሁ
18	በዚህ ሆስፒታል/ጤና ጣቢያ የሚሰጠው ህክምና የአእምሮ ህመሜ እንዳያገረሽ እየረዳኝ ነው፡፡	በፍጹም አልስማማም
		አልስማማም
		እስማማለሁ
		በጣም እስማማለሁ
19	በዚህ ሆስፒታል/ጤና ጣቢያ የሚሰጠው ህክምና ሥርቼ ገቢዬ እንዲሻሻል ረድቶኛል ፡፡	በፍጹም አልስማማም
		አልስማማም
		እስማማለሁ
		በጣም እስማማለሁ
20	በዚህ ሆስፒታል/ጤና ጣቢያ የሐኪም እርዳታ በምፈልግበት ጊዜ ሁሉ ማግኘት እችላለሁ፡፡	በፍጹም አልስማማም
		አልስማማም
		እስማማለሁ
		በጣም እስማማለሁ
21	ወደ ሆስፒታሉ/ጤና ጣቢያው ለመምጣት ቀላል ነበር።	በፍጹም አልስማማም
		አልስማማም
		እስማማለሁ
		በጣም እስማማለሁ
22	በሆስፒታሉ/ጤና ጣቢያው ተገኝቶ ለመታከም በቂ ጊዜ ነበረኝ፡፡	በፍጹም አልስማማም
		አልስማማም
		እስማማለሁ
		በጣም እስማማለሁ
23	ወደ ሆስፒታል/ጤና ጣቢያ መጥቶ ለመታከም በቂ ገንዘብ ነበረኝ፡፡	በፍጹም አልስማማም
		አልስማማም
		እስማማለሁ
		በጣም እስማማለሁ
24	ቤተሰቦቼ ወይም ጓደኞቼ ተመሳሳይ የጤና እንክብካቤ/እርዳታ ቢያስፈልጋቸው ይህንን አገልግሎት እንዲጠቀሙ እመክራለሁ፡፡	በፍጹም አልስማማም
		አልስማማም
		እስማማለሁ
		በጣም እስማማለሁ
